# Sparse Poisson regression via mixed-integer optimization

**DOI:** 10.1371/journal.pone.0249916

**Published:** 2021-04-22

**Authors:** Hiroki Saishu, Kota Kudo, Yuichi Takano

**Affiliations:** 1 Graduate School of Science and Technology, University of Tsukuba, Tsukuba, Ibaraki, Japan; 2 Faculty of Engineering, Information and Systems, University of Tsukuba, Tsukuba, Ibaraki, Japan; HEC Montréal, CANADA

## Abstract

We present a mixed-integer optimization (MIO) approach to sparse Poisson regression. The MIO approach to sparse linear regression was first proposed in the 1970s, but has recently received renewed attention due to advances in optimization algorithms and computer hardware. In contrast to many sparse estimation algorithms, the MIO approach has the advantage of finding the best subset of explanatory variables with respect to various criterion functions. In this paper, we focus on a sparse Poisson regression that maximizes the weighted sum of the log-likelihood function and the *L*_2_-regularization term. For this problem, we derive a mixed-integer quadratic optimization (MIQO) formulation by applying a piecewise-linear approximation to the log-likelihood function. Optimization software can solve this MIQO problem to optimality. Moreover, we propose two methods for selecting a limited number of tangent lines effective for piecewise-linear approximations. We assess the efficacy of our method through computational experiments using synthetic and real-world datasets. Our methods provide better log-likelihood values than do conventional greedy algorithms in selecting tangent lines. In addition, our MIQO formulation delivers better out-of-sample prediction performance than do forward stepwise selection and *L*_1_-regularized estimation, especially in low-noise situations.

## Introduction

A count variable, which takes only on nonnegative integer values, reflects the number of occurrences of an event during a fixed time period. Count regression models such as Poisson, overdispersed Poisson, and negative binomial regression are standard methods for predicting such count variables [1–3]. In particular, Poisson regression is most commonly used for count regression. There are numerous applications of Poisson regression models for predicting count variables, including manufacturing defects [4], disease incidence [5], crowd counting [6], length of hospital stay [7], and vehicle crashes [8].

The aim of sparse estimation is to decrease the number of nonzero estimates of regression coefficients. This method is often used for selecting a significant subset of explanatory variables [9–12]. Subset selection provides the following benefits:

- data collection and storage costs can be reduced,
- computational load of estimating regression coefficients can be reduced,
- interpretability of regression analysis can be increased, and
- generalization performance of a regression model can be improved.

A direct way of *best* sparse estimation involves evaluating all possible subset regression models. However, the exhaustive search method [13–15] is often computationally infeasible because the number of possible subsets grows exponentially with the number of candidate variables. In contrast, stepwise selection [15, 16], which repeats addition and elimination of one explanatory variable at a time, is a practical method for sparse estimation. Several metaheuristic algorithms have been applied to subset selection for Poisson regression [17, 18], and various regularization methods have been recently proposed for sparse Poisson regression [19–22]. Note, however, that these (non-exhaustive) sparse estimation methods are heuristic algorithms, which cannot verify optimality of an obtained subset of explanatory variables (e.g., in the maximum likelihood sense).

In this paper, we focus on the mixed-integer optimization (MIO) approach to sparse estimation. This approach was first proposed for sparse linear regression in the 1970s [23], but has recently received renewed attention due to advances in optimization algorithms and computer hardware [24–29]. In contrast to many sparse estimation algorithms, the MIO approach has the advantage of finding the best subset of explanatory variables with respect to various criterion functions, including Mallows’ *C*_*p*_ [30], adjusted *R*^2^ [31], information criteria [31–33], mRMR [34], and the cross-validation criterion [35]. MIO-based sparse estimation methods can be extended to binary or ordinal classification models [36–40] and to eliminating multicollinearity [41–44].

The log-likelihood to be maximized is a concave but nonlinear function, making it hard to apply an MIO approach to sparse Poisson regression. To remedy such nonlinearity, prior studies made effective use of piecewise-linear approximations of the log-likelihood functions, thereby yielding mixed-integer linear optimization (MILO) formulations for binary or ordinal classification [38–40]. Optimization software can solve the resultant MILO problems to optimality. Greedy algorithms for selecting a limited number of linear functions for piecewise-linear approximations have also been developed [38, 40].

This paper aims at establishing an effective MIO approach to sparse Poisson regression based on piecewise-linear approximations. Specifically, we consider a sparse Poisson regression that maximizes the weighted sum of the log-likelihood function and the *L*_2_-regularization term. To that end, we derive a mixed-integer quadratic optimization (MIQO) formulation by applying a piecewise-linear approximation to the log-likelihood function. We also propose two methods for selecting a limited number of tangent lines to improve the quality of piecewise-linear approximations.

We assess the efficacy of our method through computational experiments using synthetic and real-world datasets. Our methods for selecting tangent lines produce better log-likelihood values than do conventional greedy algorithms. For synthetic datasets, our MIQO formulation realizes better out-of-sample prediction performance than do forward stepwise selection and *L*_1_-regularized estimation, especially in low-noise situations. For real-world datasets, our MIQO formulation compares favorably with the other methods in out-of-sample prediction performance.

### Notation

Throughout this paper, sets of consecutive integers ranging from 1 to *n* are denoted as

$$\begin{array}{c} \left[n\right]:=\{\begin{array}{cc} \left\{1,2,\ldots ,n\right\} & \text{if}\;n\geq 1,\\ \emptyset & \text{otherwise}. \end{array} \end{array}$$

## Methods

This section starts with a brief review of Poisson regression, and then presents our MIO formulations for sparse Poisson regression based on piecewise-linear approximations. We then describe our methods for selecting tangent lines suitable for piecewise-linear approximations.

### Poisson regression model

Suppose we are given a sample of *n* data instances (***x***_*i*_, *y*_*i*_) for *i* ∈ [*n*], where ***x***_*i*_ ≔ (*x*_*i*1_, *x*_*i*2_, …, *x*_*ip*_)^⊤^ is a vector composed of *p* explanatory variables, and *y*_*i*_ ∈ {0}∪[*m*] is a count variable to be predicted for each instance *i* ∈ [*n*]. We define binary labels as

$$\begin{array}{c} \delta_{ik}:=\{\begin{array}{cc} 1 & \text{if}\;y_{i}=k,\\ 0 & \text{otherwise} \end{array}\;\left(i\in \left[n\right],\;k\in \left\{0\right\}\cup \left[m\right]\right). \end{array}$$ (1)

The random count variable *Y* is assumed to follow the Poisson distribution

$$\begin{array}{c} \text{Pr}\left(Y=k|\lambda \right)=\frac{\lambda^{k}\exp\left(-\lambda \right)}{k!}\;\left(k=0,1,2,\ldots \right), \end{array}$$ (2)

where $\lambda \in R_{+}$ is a parameter representing both the mean and variance of the Poisson distribution. The distribution parameter $\lambda_{i}\in R_{+}$ is explained by the linear regression model

$$\begin{array}{c} \log\lambda_{i}=w^{⊤}x_{i}+b=w_{1}x_{i1}+w_{2}x_{i2}+\cdots +w_{p}x_{ip}+b\;\left(i\in \left[n\right]\right), \end{array}$$ (3)

where ***w*** ≔ (*w*_1_, *w*_2_, …, *w*_*p*_)^⊤^ is a vector of regression coefficients, and *b* is an intercept term. Then, the occurrence probability of the given sample is expressed as

$$\begin{array}{c} \prod_{i=1}^{n}\text{Pr}\left(Y=y_{i}|\lambda_{i}\right)=\prod_{i=1}^{n}\prod_{k=0}^{m}\text{Pr}{\left(Y=k|\lambda_{i}\right)}^{\delta_{ik}}.\;∵\text{Eq}.\;\left(1\right) \end{array}$$

The regression parameters (*b*, ***w***) are estimated by maximizing the log-likelihood function

$$\begin{array}{cc} L(b,w) & ≔\log(\prod_{i=1}^{n}\prod_{k=0}^{m}\text{Pr}{\left(Y=k|\lambda_{i}\right)}^{\delta_{ik}})\\ & =\sum_{i=1}^{n}\sum_{k=0}^{m}\delta_{ik}(k\log\lambda_{i}-\lambda_{i}-\log k!)\;∵\text{Eq}.\;\left(2\right)\\ & =\sum_{i=1}^{n}\sum_{k=0}^{m}\delta_{ik}f_{k}\left(w^{⊤}x_{i}+b\right),\;∵\text{Eq}.\;\left(3\right) \end{array}$$ (4)

where *f*_*k*_(*u*) is a nonlinear function defined as

$$\begin{array}{c} f_{k}\left(u\right)=ku-\exp\left(u\right)-\log k!\;\left(k\in \left\{0\right\}\cup \left[m\right]\right). \end{array}$$ (5)

**Fig 1:**
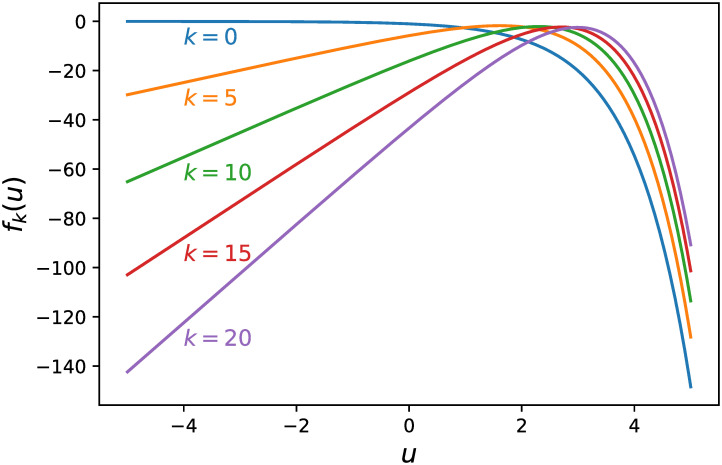
Graphs of *f*_*k*_(*u*) for *k* ∈ {0, 5, 10, 15, 20}.

Fig 1 shows graphs of *f*_*k*_(*u*) for *k* ∈ {0, 5, 10, 15, 20}. Since its second derivative $f_{k}^{\prime \prime }\left(u\right)=-\exp\left(u\right)$ is always negative, *f*_*k*_(*u*) is a nonlinear concave function.

The following theorem gives an asymptote of *f*_*k*_(*u*).

**Theorem 1**. When *u* goes to −∞, *f*_*k*_(*u*) has the asymptote

$$\begin{array}{c} \phi_{k}\left(u\right)=ku-\log k!\;\left(k\in \left\{0\right\}\cup \left[m\right]\right). \end{array}$$ (6)

*Proof*. We have

$$\begin{array}{cc} & \lim_{u\to -\infty }\frac{f_{k}\left(u\right)}{u}=\lim_{u\to -\infty }(k-\frac{\exp(u)}{u}-\frac{\log k!}{u})=k,\\ & \lim_{u\to -\infty }\left(f_{k}\left(u\right)-ku\right)=\lim_{u\to -\infty }\left(-\exp\left(u\right)-\log k!\right)=-\log k!, \end{array}$$

which completes the proof.

### Mixed-integer nonlinear optimization formulation

Before deriving our desired formulation, we introduce a mixed-integer nonlinear optimization (MINLO) formulation for sparse Poisson regression. Let *z* ≔ (*z*_1_, *z*_2_, …, *z*_*p*_)^⊤^ be a vector composed of binary decision variables for subset selection, namely,

$$\begin{array}{c} z_{j}=\{\begin{array}{cc} 1 & \text{if}\;\text{the}\;j\text{th}\;\text{explanatory}\;\text{variable}\;\text{is}\;\text{selected},\\ 0 & \text{otherwise}\;(i.e.,\;w_{j}=0) \end{array}\;\left(j\in \left[p\right]\right). \end{array}$$

To improve the generalization performance of a resultant regression model, we also introduce the *L*_2_-regularization term *α**w***^⊤^***w*** to be minimized, where $\alpha \in R_{+}$ is a user-defined regularization parameter [45]. We therefore address maximizing the weighted sum of the log-likelihood function of Eq (4) and the *L*_2_-regularization term. This sparse Poisson regression can be formulated as the MINLO problem

$$\begin{array}{cc} \underset{}{\text{maximize}} & \;\sum_{i=1}^{n}\sum_{k=0}^{m}\delta_{ik}f_{k}\left(w^{⊤}x_{i}+b\right)-\alpha w^{⊤}w \end{array}$$ (7)

$$\begin{array}{cc} \text{subject}\;\text{to} & \;z_{j}=0\;\Rightarrow \;w_{j}=0\;\left(j\in \left[p\right]\right), \end{array}$$ (8)

$$\begin{array}{cc} & \;\sum_{j=1}^{p}z_{j}=\theta , \end{array}$$ (9)

$$\begin{array}{cc} & \;b\in R,\;w\in R^{p},\;z\in {\left\{0,1\right\}}^{p}, \end{array}$$ (10)

where *θ* ∈ [*p*] is a user-defined parameter of the subset size. If *z*_*j*_ = 0, then the *j*th coefficient must be zero by logical implication of Eq (8). Eq (9) specifies the number of nonzero regression coefficients, and Eq (10) lists all decision variables.

The logical implication of Eq (8) can be imposed by using indicator constraints implemented in modern optimization software. Eq (8) can also be represented as

$$\begin{array}{c} -Mz_{j}\leq w_{j}\leq Mz_{j}\;\left(j\in \left[p\right]\right), \end{array}$$

where $M\in R_{+}$ is a sufficiently large positive constant.

### Piecewise-linear approximation

It is very difficult to handle the MINLO problem by Eqs (7)–(10) using MIO software, because Eq (7) to be maximized is a concave but nonlinear function. Following prior studies [38–40], we apply piecewise-linear approximation techniques to the nonlinear function of Eq (5).

Letting {(*u*_*kℓ*_, *f*_*k*_(*u*_*kℓ*_))∣*ℓ* ∈ [*h*]} be a set of *h* tangent points for the function *f*_*k*_(*u*), the corresponding tangent lines are

$$\begin{array}{c} g_{k}\left(u|u_{k\ell }\right)≔f_{k}^{\prime }\left(u_{k\ell }\right)\left(u-u_{k\ell }\right)+f_{k}\left(u_{k\ell }\right)\;\left(\ell \in \left[h\right]\right), \end{array}$$ (11)

where $f_{k}^{\prime }\left(u\right)=k-\exp\left(u\right)$ is the derivative of *f*_*k*_(*u*).

**Fig 2:**
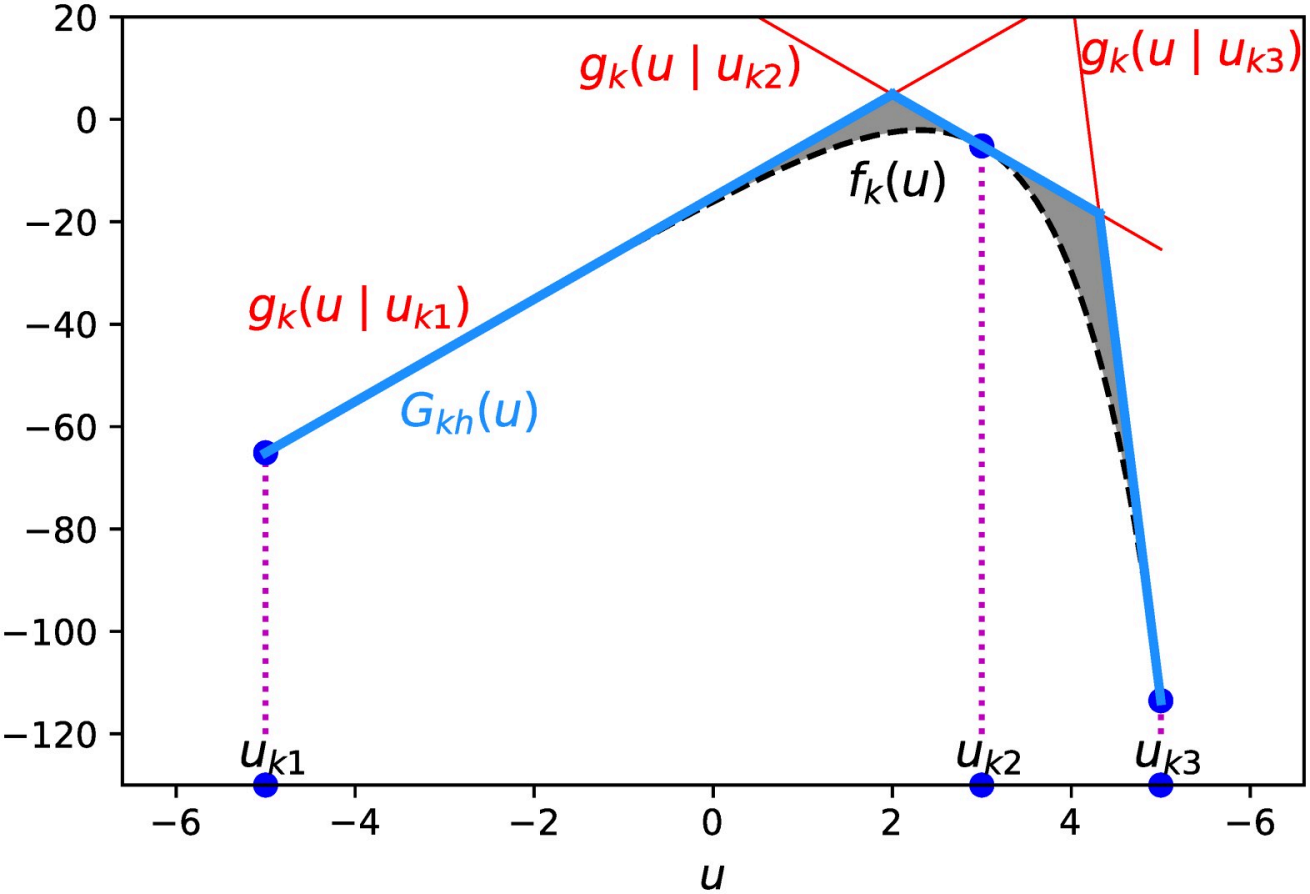
Piecewise-linear approximation of *f*_*k*_(*u*) for *k* = 10.

As Fig 2 shows, the graph of a concave function lies below its tangent lines, so *f*_*k*_(*u*) can be approximated by the pointwise minimum of a set of *h* tangent lines. For each *u*, we approximate *f*_*k*_(*u*) by

$$\begin{array}{cc} G_{kh}\left(u\right) & ≔\min\{g_{k}\left(u|u_{k\ell }\right)|\ell \in \left[h\right]\}\\ & =\max\{t|t\leq g_{k}\left(u|u_{k\ell }\right)\;\left(\ell \in \left[h\right]\right)\}, \end{array}$$ (12)

where t∈R is an auxiliary decision variable.

We next focus on the approximation gap $g_{k}\left(u|\bar{u}\right)-f_{k}\left(u\right)$ arising from a tangent point $\left(\bar{u},f_{k}\left(\bar{u}\right)\right)$. By the following theorem, this gap does not depend on *k*; therefore, we can employ the same set {*u*_*ℓ*_∣*ℓ* ∈ [*h*]} for all *k* ∈ {0}∪[*m*] when selecting tangent points for piecewise-linear approximations.

**Theorem 2**. $g_{k}\left(u|\bar{u}\right)-f_{k}\left(u\right)$ is independent of *k* ∈ {0}∪[*m*].

*Proof*. We have

$$\begin{array}{cc} & g_{k}\left(u|\bar{u}\right)-f_{k}\left(u\right)\\ =\; & \left(k-\exp\left(\bar{u}\right)\right)\left(u-\bar{u}\right)+k\bar{u}-\exp\left(\bar{u}\right)-\log k!-\left(ku-\exp\left(u\right)-\log k!\right)\\ =\; & -\exp\left(\bar{u}\right)\left(u-\bar{u}\right)-\exp\left(\bar{u}\right)+\exp\left(u\right), \end{array}$$

which completes the proof.

### Mixed-integer quadratic optimization formulation

We are now ready to present our desired formulation for sparse Poisson regression. Let ***T*** ≔ (*t*_*ik*_)_(*i*, *k*)∈[*n*] × ({0}∪[*m*])_ be a matrix composed of auxiliary decision variables for piecewise-linear approximations. We substitute Eq (11) and *u* = ***w***^⊤^***x***_*i*_ + *b* into Eq (12) to make a piecewise-linear approximation of the objective function of Eq (7). By Theorem 2, we use {(*u*_*ℓ*_, *f*_*k*_(*u*_*ℓ*_))∣*ℓ* ∈ [*h*]} as a set of *h* tangent points for the function *f*_*k*_(*u*). Consequently, the MINLO problem by Eqs (7)–(10) can be reduced to the MIQO problem

$$\begin{array}{cc} \underset{}{\text{maximize}} & \;\sum_{i=1}^{n}\sum_{k=0}^{m}\delta_{ik}t_{ik}-\alpha w^{⊤}w \end{array}$$ (13)

$$\begin{array}{cc} \text{subject}\;\text{to} & \;t_{ik}\leq f_{k}^{\prime }\left(u_{\ell }\right)\left(w^{⊤}x_{i}+b-u_{\ell }\right)+f_{k}\left(u_{\ell }\right)\\ & (i\in [n],\;k\in \{0\}\cup [m],\;\ell \in [h]), \end{array}$$ (14)

$$\begin{array}{cc} & \;z_{j}=0\;\Rightarrow \;w_{j}=0\;\left(j\in \left[p\right]\right), \end{array}$$ (15)

$$\begin{array}{cc} & \;\sum_{j=1}^{p}z_{j}=\theta , \end{array}$$ (16)

$$\begin{array}{cc} & \;b\in R,\;w\in R^{p},\;T\in R^{n\times (m+1)},\;z\in {\left\{0,1\right\}}^{p}, \end{array}$$ (17)

where Eq (17) lists all of the decision variables. Note that optimization software can solve this MIQO problem to optimality.

### Previous algorithms for selecting tangent lines

The accuracy of piecewise-linear approximations depends on the associated set of tangent lines. It is clear that with increasingly many appropriate tangent lines, the MIQO problem by Eqs (13)–(17) approaches the original MINLO problem by Eqs (7)–(10). In this case, however, solving the MIQO problem becomes computationally expensive because the problem size grows larger. It is therefore crucial to limit the number of tangent lines for effective approximations.

Sato et al. [40] developed a greedy algorithm for selecting tangent lines to approximate the logistic loss function. This algorithm adds tangent lines one by one so that the total approximation gap (the area of the shaded portion in Fig 2) will be minimized. Naganuma et al. [38] employed a greedy algorithm that selects tangent planes to approximate the bivariate nonlinear function for ordinal classification. This algorithm iteratively selects tangent points where the approximation gap is largest.

These previous algorithms have two limitations addressed in this paper. First, they totally ignore the properties of the sample distribution. Second, tangent lines are determined one at a time, so the resultant set of tangent lines is not necessarily optimal. In the following sections, we propose two methods, namely the adaptive greedy algorithm and the simultaneous optimization method, to resolve the first and second limitations, respectively.

### Adaptive greedy algorithm

Our first method, the *adaptive greedy algorithm*, selects tangent lines depending on the sample distribution.

Suppose we are given $\left(\bar{b},\bar{w}\right)$ as regression parameter values. These values can be obtained, for example, through maximum likelihood estimation of the full model of Eq (3). We then have an empirical distribution of input values for the nonlinear function of Eq (5) as ${\bar{u}}_{i}≔{\bar{w}}^{⊤}x_{i}+\bar{b}$ for *i* ∈ [*n*]. Our algorithm aims to minimize the sum of squared approximation gaps in response to this empirical distribution. Although the previous algorithms compute a set of tangent lines independent of datasets, our algorithm can adapt a set of tangent lines to each dataset.

We select *h* tangent points $u_{1}^{*},u_{2}^{*},\ldots ,u_{h}^{*}$ sequentially, where the *s*th tangent point $u_{s}^{*}$ is determined on the condition that previous tangent points $u_{1}^{*},u_{2}^{*},\ldots ,u_{s-1}^{*}$ are fixed. This stepwise greedy procedure is formulated as

$$\begin{array}{c} u_{s}^{*}\in \underset{u_{s}\in R}{\text{arg}\;\text{min}}\{\sum_{i=1}^{n}{\left(G_{ks}\left({\bar{u}}_{i}\right)-f_{k}\left({\bar{u}}_{i}\right)\right)}^{2}\;|\begin{array}{c} u_{\ell }=u_{\ell }^{*}\;\left(\ell \in \left[s-1\right]\right)\\ L\leq u_{s}\leq U \end{array}\}\;\left(s\in \left[h\right]\right), \end{array}$$ (18)

where *G*_*ks*_(*u*) = min{*g*_*k*_(*u*∣*u*_*ℓ*_)∣*ℓ*∈[*s*]}, and [*L*, *U*] is an input interval of the nonlinear function of Eq (5). Notably, by Theorem 2 this algorithm yields the same set of tangent lines for all *k* ∈ {0}∪[*m*].

### Simultaneous optimization method

Our second method, the *simultaneous optimization method*, selects a set of *h* tangent lines simultaneously, not sequentially.

Suppose the intersection between the *ℓ*th and (*ℓ* + 1)th tangent lines is specified by *c*_*k*_(*u*_*ℓ*_, *u*_*ℓ*+1_), meaning *g*_*k*_(*u*∣*u*_*ℓ*_) = *g*_*k*_(*u*∣*u*_*ℓ*+1_) holds when *u* = *c*_*k*_(*u*_*ℓ*_, *u*_*ℓ*+1_). It follows from Eq (11) that

$$\begin{array}{c} c_{k}\left(u_{\ell },u_{\ell +1}\right)=\frac{f_{k}^{\prime }\left(u_{\ell }\right)u_{\ell }-f_{k}^{\prime }\left(u_{\ell +1}\right)u_{\ell +1}+f_{k}\left(u_{\ell +1}\right)-f_{k}\left(u_{\ell }\right)}{f_{k}^{\prime }\left(u_{\ell }\right)-f_{k}^{\prime }\left(u_{\ell +1}\right)}\;\left(\ell \in \left[h-1\right]\right). \end{array}$$ (19)

We then simultaneously determine a set of *h* tangent points minimizing the total approximation gap (the area of the shaded portion in Fig 2). This procedure can be posed as the nonlinear optimization (NLO) problem

$$\begin{array}{cc} \underset{}{\text{minimize}} & \;\sum_{\ell =1}^{h}\int_{c_{k}\left(u_{\ell -1},u_{\ell }\right)}^{c_{k}\left(u_{\ell },u_{\ell +1}\right)}\left(g_{k}\left(u|u_{\ell }\right)-f_{k}\left(u\right)\right)\;du \end{array}$$ (20)

$$\begin{array}{cc} \text{subject}\;\text{to} & \;L\leq u_{1}\leq u_{2}\leq \cdots \leq u_{h}\leq U, \end{array}$$ (21)

$$\begin{array}{cc} & \;\left(u_{1},u_{2},\ldots ,u_{h}\right)\in R^{h}, \end{array}$$ (22)

where *c*_*k*_(*u*_0_, *u*_1_) = *L* and *c*_*k*_(*u*_*h*_, *u*_*h*+1_) = *U* are fixed, and *c*_*k*_(*u*_*ℓ*_, *u*_*ℓ*+1_) is defined by Eq (19) for *ℓ* ∈ [*h* − 1]. NLO software can handle this problem, yielding a locally optimal set of tangent points. This method also provides the same set of tangent lines for all *k* ∈ {0}∪[*m*].

## Experimental results and discussion

This section describes computational experiments for evaluating the effectiveness of our method for sparse Poisson regression.

### Methods for comparison

We investigate the performance of our MIQO formulation by Eqs (13)–(17) using tangent lines selected by each of the following methods, where *h* is the number of tangent lines to be selected.

**EqlSpc**(*h*): setting equally spaced tangent points
**AreaGrd**(*h*): the greedy algorithm developed by Sato et al. [40]
**GapGrd**(*h*): the greedy algorithm developed by Naganuma et al. [38]
**AdpGrd**(*h*): our adaptive greedy algorithm by Eq (18)
**SmlOpt**(*h*): our simultaneous optimization method by Eqs (20)–(22)

We implemented these algorithms in the Python programming language. We set the input interval [*L*, *U*] = [−5, 5] and use the asymptote of Eq (6) as the initial tangent line. We use the Python statsmodels module to perform maximum likelihood estimation of the full model of Eq (3), then select tangent points of Eq (18) by evaluating each point *u*_*s*_ ∈ {−5.00, −4.99, −4.98, …, 4.99, 5.00} for *s* ∈ [*h*]. We use the Python scipy.optimize module (method=’SLSQP’) to solve the NLO problem by Eqs (20)–(22). We use Gurobi Optimizer 8.1.1 (https://www.gurobi.com/) to solve the MIQO problem by Eqs (13)–(17), and the indicator constraint to impose the logical implication of Eq (15). We fix the *L*_2_-regularization parameter to *α* = 0 in Tables 1, 2 and 6, whereas we tune it through hold-out validation using the training instances in Tables 3, 4 and 7.

**Table 1:** Results of our MIQO formulation for synthetic training instances (*θ* = 5).

*σ*^2^	*ρ*	Method	LogLkl	Time (s)	
				MIQO	TngLine
0.01	0.35	EqlSpc(10)	−119.01 (±1.57)	1.06 (±0.22)	0.00 (±0.00)
		AreaGrd(10)	−182.04 (±2.48)	0.04 (±0.00)	0.08 (±0.00)
		GapGrd(10)	−516.83 (±1.73)	0.04 (±0.00)	0.10 (±0.00)
		AdpGrd(10)	−**107.10** (±1.60)	0.28 (±0.03)	7.98 (±0.02)
		SmlOpt(10)	−137.48 (±7.51)	0.25 (±0.07)	0.02 (±0.00)
	0.70	EqlSpc(10)	−129.63 (±1.69)	7.97 (±0.96)	0.00 (±0.00)
		AreaGrd(10)	−183.20 (±1.05)	0.04 (±0.00)	0.08 (±0.00)
		GapGrd(10)	−510.43 (±2.59)	0.04 (±0.00)	0.10 (±0.00)
		AdpGrd(10)	−118.08 (±3.11)	1.49 (±0.37)	8.00 (±0.03)
		SmlOpt(10)	−**117.17** (±1.61)	3.99 (±1.25)	0.02 (±0.00)
0.10	0.35	EqlSpc(10)	−130.52 (±1.92)	1.92 (±0.52)	0.00 (±0.00)
		AreaGrd(10)	−186.59 (±3.26)	0.04 (±0.00)	0.08 (±0.00)
		GapGrd(10)	−519.94 (±3.32)	0.04 (±0.00)	0.10 (±0.00)
		AdpGrd(10)	−**112.95** (±1.99)	0.35 (±0.03)	7.94 (±0.02)
		SmlOpt(10)	−139.92 (±7.57)	0.60 (±0.26)	0.02 (±0.00)
	0.70	EqlSpc(10)	−127.65 (±2.75)	5.96 (±1.11)	0.00 (±0.00)
		AreaGrd(10)	−188.72 (±2.49)	0.04 (±0.00)	0.09 (±0.00)
		GapGrd(10)	−523.75 (±4.00)	0.04 (±0.00)	0.10 (±0.00)
		AdpGrd(10)	−**124.06** (±4.85)	1.87 (±0.45)	7.96 (±0.03)
		SmlOpt(10)	−131.86 (±6.76)	2.84 (±0.85)	0.02 (±0.00)
1.00	0.35	EqlSpc(10)	−173.40 (±5.81)	3.39 (±0.89)	0.00 (±0.00)
		AreaGrd(10)	−208.61 (±3.79)	0.04 (±0.00)	0.08 (±0.00)
		GapGrd(10)	−519.65 (±5.60)	0.04 (±0.00)	0.10 (±0.00)
		AdpGrd(10)	−**148.60** (±3.35)	1.95 (±0.31)	8.01 (±0.01)
		SmlOpt(10)	−172.29 (±7.08)	1.26 (±0.47)	0.02 (±0.00)
	0.70	EqlSpc(10)	−194.70 (±19.21)	7.48 (±1.75)	0.00 (±0.00)
		AreaGrd(10)	−214.68 (±3.99)	0.04 (±0.00)	0.08 (±0.00)
		GapGrd(10)	−516.71 (±5.43)	0.04 (±0.00)	0.10 (±0.00)
		AdpGrd(10)	−**159.21** (±5.81)	4.29 (±1.30)	8.05 (±0.05)
		SmlOpt(10)	−165.46 (±5.47)	2.95 (±0.79)	0.02 (±0.00)

**Table 2:** Results of our MIQO formulation for synthetic training instances (*θ* = 10).

*σ*^2^	*ρ*	Method	LogLkl	Time (s)	
				MIQO	TngLine
0.01	0.35	EqlSpc(10)	−105.00 (±0.62)	0.36 (±0.01)	0.00 (±0.00)
		AreaGrd(10)	−105.16 (±0.78)	0.48 (±0.06)	0.23 (±0.00)
		GapGrd(10)	−106.69 (±0.84)	0.54 (±0.07)	0.53 (±0.00)
		AdpGrd(10)	−**102.25** (±0.53)	0.40 (±0.01)	18.46 (±0.03)
		SmlOpt(10)	−103.99 (±0.63)	0.39 (±0.02)	0.08 (±0.00)
	0.70	EqlSpc(10)	−107.37 (±0.96)	2.37 (±0.88)	0.00 (±0.00)
		AreaGrd(10)	−109.83 (±0.74)	5.03 (±1.26)	0.23 (±0.00)
		GapGrd(10)	−111.34 (±1.04)	3.98 (±0.79)	0.53 (±0.00)
		AdpGrd(10)	−**105.22** (±0.86)	0.55 (±0.06)	18.48 (±0.03)
		SmlOpt(10)	−107.78 (±1.02)	3.44 (±1.09)	0.08 (±0.00)
0.10	0.35	EqlSpc(10)	−109.65 (±1.19)	0.47 (±0.03)	0.00 (±0.00)
		AreaGrd(10)	−110.51 (±1.16)	0.65 (±0.06)	0.24 (±0.00)
		GapGrd(10)	−113.05 (±0.59)	1.06 (±0.17)	0.53 (±0.00)
		AdpGrd(10)	−**107.30** (±1.26)	0.46 (±0.02)	18.46 (±0.03)
		SmlOpt(10)	−108.81 (±1.27)	0.55 (±0.05)	0.08 (±0.00)
	0.70	EqlSpc(10)	−108.93 (±1.37)	2.98 (±0.92)	0.00 (±0.00)
		AreaGrd(10)	−110.82 (±1.42)	6.33 (±1.00)	0.23 (±0.00)
		GapGrd(10)	−112.60 (±1.32)	5.28 (±1.12)	0.52 (±0.00)
		AdpGrd(10)	−**106.20** (±1.17)	1.31 (±0.25)	18.44 (±0.04)
		SmlOpt(10)	−107.96 (±1.29)	3.55 (±0.69)	0.08 (±0.00)
1.00	0.35	EqlSpc(10)	−148.55 (±4.03)	4.61 (±1.57)	0.00 (±0.00)
		AreaGrd(10)	−150.45 (±3.75)	5.88 (±1.99)	0.23 (±0.00)
		GapGrd(10)	−155.41 (±3.54)	2.98 (±0.86)	0.52 (±0.01)
		AdpGrd(10)	−**146.51** (±3.84)	3.52 (±1.76)	18.50 (±0.03)
		SmlOpt(10)	−148.41 (±3.88)	4.35 (±1.52)	0.08 (±0.00)
	0.70	EqlSpc(10)	−151.37 (±3.67)	6.38 (±1.43)	0.00 (±0.00)
		AreaGrd(10)	−153.25 (±3.56)	8.58 (±1.41)	0.23 (±0.00)
		GapGrd(10)	−154.34 (±4.24)	4.21 (±0.90)	0.53 (±0.00)
		AdpGrd(10)	−**149.30** (±3.55)	6.48 (±0.78)	18.47 (±0.04)
		SmlOpt(10)	−150.80 (±3.51)	6.37 (±1.04)	0.08 (±0.00)

**Table 3:** Prediction performance for synthetic test instances (*θ* = 5).

*σ*^2^	*ρ*	Method	RMSE	Accuracy	Recall	Time (s)
0.01	0.35	AdpGrd(30)	1.337 (±0.029)	0.430 (±0.004)	**0.500** (±0.000)	494.80 (±8.10)
		SmlOpt(30)	**1.330** (±0.033)	**0.435** (±0.005)	**0.500** (±0.000)	53.63 (±4.12)
		FwdStep	2.040 (±0.017)	0.366 (±0.002)	0.480 (±0.042)	0.68 (±0.02)
		L1-Rgl	2.012 (±0.016)	0.367 (±0.002)	0.480 (±0.042)	0.87 (±0.01)
	0.70	AdpGrd(30)	1.167 (±0.046)	**0.463** (±0.011)	0.420 (±0.079)	732.13 (±26.12)
		SmlOpt(30)	**1.158** (±0.041)	**0.463** (±0.011)	**0.440** (±0.084)	227.06 (±18.01)
		FwdStep	1.987 (±0.020)	0.388 (±0.001)	0.400 (±0.067)	0.65 (±0.01)
		L1-Rgl	1.959 (±0.015)	0.384 (±0.004)	0.000 (±0.133)	0.89 (±0.02)
0.10	0.35	AdpGrd(30)	1.523 (±0.048)	0.413 (±0.005)	**0.500** (±0.000)	500.26 (±9.34)
		SmlOpt(30)	**1.515** (±0.052)	**0.416** (±0.005)	**0.500** (±0.000)	55.73 (±5.70)
		FwdStep	2.090 (±0.029)	0.361 (±0.004)	0.490 (±0.032)	0.65 (±0.02)
		L1-Rgl	2.037 (±0.021)	0.363 (±0.004)	0.460 (±0.052)	0.92 (±0.01)
	0.70	AdpGrd(30)	1.423 (±0.100)	0.433 (±0.008)	0.450 (±0.071)	681.68 (±31.72)
		SmlOpt(30)	**1.402** (±0.093)	**0.438** (±0.009)	**0.470** (±0.048)	202.56 (±19.11)
		FwdStep	2.086 (±0.065)	0.384 (±0.003)	0.390 (±0.074)	0.71 (±0.02)
		L1-Rgl	2.022 (±0.021)	0.378 (±0.002)	0.300 (±0.105)	1.02 (±0.03)
1.00	0.35	AdpGrd(30)	2.201 (±0.076)	**0.334** (±0.009)	**0.400** (±0.094)	500.35 (±7.52)
		SmlOpt(30)	2.209 (±0.075)	0.330 (±0.010)	0.390 (±0.099)	56.51 (±4.62)
		FwdStep	2.218 (±0.074)	0.333 (±0.009)	0.390 (±0.099)	0.93 (±0.05)
		L1-Rgl	**2.133** (±0.045)	0.329 (±0.009)	0.340 (±0.097)	1.03 (±0.02)
	0.70	AdpGrd(30)	2.188 (±0.083)	**0.361** (±0.004)	**0.310** (±0.099)	587.62 (±29.25)
		SmlOpt(30)	2.198 (±0.094)	**0.361** (±0.006)	**0.310** (±0.074)	121.24 (±17.89)
		FwdStep	2.173 (±0.052)	0.360 (±0.005)	0.290 (±0.088)	0.83 (±0.05)
		L1-Rgl	**2.057** (±0.032)	0.357 (±0.006)	0.250 (±0.071)	1.06 (±0.03)

**Table 4:** Prediction performance for synthetic test instances (*θ* = 10).

*σ*^2^	*ρ*	Method	RMSE	Accuracy	Recall	Time (s)
0.01	0.35	AdpGrd(30)	**0.524** (±0.042)	**0.502** (±0.019)	**1.000** (±0.000)	455.61 (±2.96)
		SmlOpt(30)	0.566 (±0.055)	0.492 (±0.018)	**1.000** (±0.000)	38.42 (±2.97)
		FwdStep	0.644 (±0.059)	0.490 (±0.018)	0.980 (±0.013)	0.67 (±0.02)
		L1-Rgl	0.908 (±0.043)	0.474 (±0.010)	0.910 (±0.028)	0.08 (±0.00)
	0.70	AdpGrd(30)	0.497 (±0.032)	0.520 (±0.029)	**1.000** (±0.000)	1664.84 (±225.86)
		SmlOpt(30)	**0.490** (±0.024)	**0.526** (±0.032)	**1.000** (±0.000)	1166.14 (±184.21)
		FwdStep	0.733 (±0.053)	0.497 (±0.020)	0.870 (±0.021)	0.73 (±0.02)
		L1-Rgl	0.885 (±0.040)	0.479 (±0.015)	0.620 (±0.055)	0.07 (±0.00)
0.10	0.35	AdpGrd(30)	**0.888** (±0.021)	**0.492** (±0.022)	**1.000** (±0.000)	468.09 (±6.20)
		SmlOpt(30)	0.911 (±0.022)	0.487 (±0.017)	**1.000** (±0.000)	40.94 (±4.13)
		FwdStep	1.147 (±0.157)	0.461 (±0.016)	0.990 (±0.010)	0.70 (±0.04)
		L1-Rgl	1.169 (±0.103)	0.444 (±0.011)	0.890 (±0.028)	0.07 (±0.00)
	0.70	AdpGrd(30)	**1.087** (±0.137)	**0.479** (±0.013)	**0.940** (±0.031)	1742.37 (±354.82)
		SmlOpt(30)	1.144 (±0.142)	0.467 (±0.011)	0.930 (±0.033)	959.33 (±230.95)
		FwdStep	1.312 (±0.158)	0.446 (±0.007)	0.820 (±0.025)	0.71 (±0.02)
		L1-Rgl	1.169 (±0.039)	0.455 (±0.008)	0.610 (±0.043)	0.07 (±0.00)
1.00	0.35	AdpGrd(30)	2.342 (±0.145)	**0.356** (±0.006)	**0.700** (±0.030)	584.74 (±35.61)
		SmlOpt(30)	2.378 (±0.153)	0.352 (±0.006)	0.690 (±0.031)	100.76 (±19.78)
		FwdStep	2.293 (±0.096)	**0.356** (±0.006)	0.690 (±0.041)	0.86 (±0.04)
		L1-Rgl	**2.133** (±0.055)	0.352 (±0.008)	0.610 (±0.043)	0.07 (±0.00)
	0.70	AdpGrd(30)	2.530 (±0.096)	0.354 (±0.005)	0.460 (±0.022)	804.62 (±72.09)
		SmlOpt(30)	2.457 (±0.086)	0.356 (±0.004)	0.470 (±0.026)	296.92 (±52.32)
		FwdStep	2.307 (±0.067)	0.363 (±0.004)	0.540 (±0.027)	0.84 (±0.05)
		L1-Rgl	**2.097** (±0.040)	**0.375** (±0.003)	**0.550** (±0.027)	0.07 (±0.00)

We compare the performance of our method with the following sparse estimation algorithms:

**FwdStep**: forward stepwise Poisson regression [15, 16]
**L1-Rgl**: *L*_1_-regularized Poisson regression [46]

We implemented these algorithms using the step function and the glmnet package [46] in the R programming language. We tune the *L*_1_-regularization parameter such that the number of nonzero regression coefficients equals *θ*, then select the corresponding subset of explanatory variables. All computations occurred on a Windows computer with an Intel Core i3-8100 CPU (3.50 GHz) and 8 GB of memory.

We use the following evaluation metrics to compare the performance of sparse estimation methods. Let ${\hat{\lambda }}_{i}$ be a predicted value based on Eq (3) for *i* ∈ *N*, where *N* is the index set of test instances. We then set ${\hat{k}}_{i}=\left\lfloor {\hat{\lambda }}_{i}\right\rfloor \in \underset{k=0,1,2,\ldots }{\text{arg}\;\text{max}}\text{Pr}\left(Y=k|{\hat{\lambda }}_{i}\right)$ based on Eq (2) for *i* ∈ *N*. The magnitude of out-of-sample prediction errors is

$$\begin{array}{c} \text{RMSE}≔\sqrt{\frac{1}{\left|N\right|}\sum_{i\in N}{\left(y_{i}-{\hat{\lambda }}_{i}\right)}^{2}}, \end{array}$$

and the number of correct class labels is

$$\begin{array}{c} \text{Accuracy}≔\frac{\left|\{i\in N|y_{i}={\hat{k}}_{i}\}\right|}{\left|N\right|}. \end{array}$$

Let S* and $\hat{S}$ respectively be true and selected subsets of explanatory variables. Note that the true subset of Eq (23) is specified for only synthetic datasets. The accuracy of subset selection is quantified as

$$\begin{array}{c} \text{Recall}≔\frac{|S^{*}\cap \hat{S}|}{|S^{*}|}. \end{array}$$

### Experimental design for synthetic datasets

Following prior studies [24, 26], we prepared synthetic datasets via the following steps. Here, we set the number of candidate explanatory variables as *p* = 30 and the maximum value of the count variable as *m* = 10.

First, we defined a vector of true regression coefficients as

$$\begin{array}{cc} w^{*} & ≔{\left(1,0,0,1,0,0,1,0,0,\ldots ,1,0,0\right)}^{⊤}\in R^{30},\\ S^{*} & ≔\left\{1,4,7\ldots ,28\right\}\;(\text{i.e.,}\;|S^{*}|=10). \end{array}$$ (23)

We next sampled explanatory variables from a normal distribution as ***x***_*i*_ ∼ N(**0**, **Σ**), where $\Sigma \in R^{30\times 30}$ is the covariance matrix. The (*i*, *j*)th entry of **Σ** is *ρ*^|*i* − *j*|^, where *ρ* represents the correlation strength between explanatory variables. We also sampled the error term from a normal distribution as *ε*_*i*_ ∼ N(0, *σ*^2^), where *σ* is the standard deviation. We then generated the count variable *y*_*i*_ ∈ {0}∪[10] by rounding

$$\begin{array}{c} \exp(\frac{{\left(w^{*}\right)}^{⊤}x_{i}}{\sqrt{{\left(w^{*}\right)}^{⊤}\Sigma w^{*}}}+\varepsilon_{i}) \end{array}$$

to the nearest integer. We tested *ρ* ∈ {0.35, 0.70} and *σ*^2^ ∈ {0.01, 0.10, 1.00} in the experiments.

We trained sparse Poisson regression models with 100 training instances. We estimated prediction performance by applying the trained regression model to sufficiently many test instances. The tables show average values for 10 repetitions, with standard errors in parentheses.

### Results for synthetic datasets

Tables 1 and 2 show the results of our MIQO formulation for the synthetic training instances with subset sizes *θ* = 5 and 10, respectively. The column labeled “LogLkl” shows the log-likelihood value of Eq (4), which was maximized using a selected subset of explanatory variables. The largest log-likelihood values for each problem instance (*σ*^2^, *ρ*) are shown in bold. The columns labeled “Time (s)” show computation times in seconds required for solving the MIQO problem (MIQO) and for selecting tangent lines (TngLine).

Our adaptive greedy algorithm (AdpGrd) attained the largest log-likelihood values for most problem instances but required long computation times to select tangent lines. This result implies that effective sets of tangent lines are different depending on the dataset, so the adaptive greedy algorithm, which computes a different set of tangent lines suitable for each dataset, can perform well. Our simultaneous optimization method (SmlOpt), on the other hand, selected tangent lines very quickly and also provided the second-best log-likelihood values for a majority of problem instances. These results clearly show that our AdpGrd and SmlOpt methods can find sparse regression models of better quality than do the conventional AreaGrd and GapGrd methods.

Tables 3 and 4 show the prediction performance of sparse Poisson regression models for synthetic test instances with subset sizes *θ* = 5 and 10, respectively. The best RMSE, accuracy, and recall values for each problem instance (*σ*^2^, *ρ*) are shown in bold.

When *σ*^2^ ∈ {0.01, 0.10}, our AdpGrd and SmlOpt methods delivered better prediction performance than did the FwdStep and L1-Rgl algorithms for all problem instances. In contrast, L1-Rgl algorithm performed very well when (*σ*^2^, *ρ*) = (1.00, 0.70) in Table 4. These results suggest that especially in low-noise situations, our MIO-based sparse estimation methods can deliver superior prediction performance as compared with heuristic algorithms such as stepwise selection and *L*_1_-regularized estimation. This observation is consistent with the simulation results reported by Hastie et al. [26].

### Experimental design for real-world datasets

Table 5 lists real-world datasets downloaded from the UCI Machine Learning Repository [47], where *n* and *p* are numbers of data instances and candidate explanatory variables, respectively. In a preprocessing step, we divided the total number of rental bikes by *d*, rounding down to the nearest integer to be an appropriate scale for the count variable to be predicted. We transformed each categorical variable into a set of dummy variables. Note that variables “dteday,” “casual,” and “registered” are not suitable for prediction purposes and thus were removed. Data instances having outliers or missing values were eliminated.

**Table 5:** Real-world datasets.

Abbr.	*n*	*p*	*d*	Original dataset [47]
Bike-H	17,379	33	100	Bike Sharing Dataset (hour)
Bike-D	731	33	1000	Bike Sharing Dataset (day)

Training instances were randomly sampled, with 500 training instances for the Bike-H dataset and 365 for the Bike-D dataset. We used the remaining instances as test instances. The tables show averaged values for 10 trials, with standard errors in parentheses.

### Results for real-world datasets

Table 6 gives the results of our MIQO formulation for the real-world training instances with subset size *θ* ∈ {5, 10}. As with the synthetic training instances (Tables 1 and 2), our adaptive greedy algorithm AdpGrd achieved the largest log-likelihood values, but with long computation times. Our simultaneous optimization method SmlOpt was much faster than AdpGrd and provided good log-likelihood values for both the Bike-H and Bike-D datasets.

**Table 6:** Results of our MIQO formulation for real-world training instances.

Dataset	*θ*	Method	LogLkl	Time (s)	
				MIQO	TngLine
Bike-H	5	EqlSpc(10)	−744.91 (±7.70)	5.87 (±0.72)	0.00 (±0.00)
		AreaGrd(10)	−785.15 (±28.70)	6.27 (±0.75)	0.23 (±0.00)
		GapGrd(10)	−938.96 (±22.97)	1.61 (±0.59)	0.53 (±0.00)
		AdpGrd(10)	−**742.98** (±7.58)	8.23 (±0.87)	94.13 (±1.11)
		SmlOpt(10)	−745.66 (±7.70)	5.54 (±0.49)	0.08 (±0.00)
	10	EqlSpc(10)	−730.67 (±7.97)	69.47 (±23.99)	0.00 (±0.00)
		AreaGrd(10)	−739.34 (±7.82)	116.71 (±30.54)	0.23 (±0.00)
		GapGrd(10)	−896.40 (±29.85)	10.42 (±4.22)	0.53 (±0.00)
		AdpGrd(10)	−**728.35** (±7.77)	67.75 (±15.86)	93.40 (±0.86)
		SmlOpt(10)	−731.52 (±7.90)	54.56 (±13.63)	0.08 (±0.00)
Bike-D	5	EqlSpc(10)	−784.89 (±3.18)	1.55 (±0.31)	0.00 (±0.00)
		AreaGrd(10)	−795.69 (±15.86)	0.74 (±0.28)	0.23 (±0.00)
		GapGrd(10)	−755.64 (±28.97)	0.96 (±0.11)	0.54 (±0.01)
		AdpGrd(10)	−**634.00** (±17.10)	6.84 (±0.62)	71.24 (±2.39)
		SmlOpt(10)	−720.46 (±7.90)	2.32 (±0.46)	0.08 (±0.00)
	10	EqlSpc(10)	−783.87 (±3.19)	2.98 (±1.79)	0.00 (±0.00)
		AreaGrd(10)	−780.44 (±2.53)	4.35 (±4.01)	0.23 (±0.00)
		GapGrd(10)	−754.38 (±29.08)	0.50 (±0.13)	0.54 (±0.01)
		AdpGrd(10)	−**626.22** (±16.72)	123.06 (±23.66)	70.77 (±2.39)
		SmlOpt(10)	−698.47 (±14.19)	9.69 (±4.42)	0.08 (±0.00)

Table 7 shows the prediction performance of sparse Poisson regression models for the real-world test instances with subset size *θ* ∈ {5, 10}. Our AdpGrd and SmlOpt methods were superior to the FwdStep and L1-Rgl algorithms in terms of RMSE values for the Bike-H dataset and accuracy values for the Bike-D dataset. FwdStep gave the best accuracy values for the Bike-H dataset, whereas there was no clear best or worst method regarding RMSE values for the Bike-D dataset.

**Table 7:** Prediction performance for real-world test instances.

Dataset	*θ*	Method	RMSE	Accuracy	Time (s)
Bike-H	5	AdpGrd(30)	**1.491** (±0.004)	0.408 (±0.004)	2530.03 (±64.29)
		SmlOpt(30)	**1.491** (±0.004)	0.407 (±0.004)	240.69 (±31.57)
		FwdStep	1.494 (±0.005)	**0.414** (±0.002)	1.61 (±0.07)
		L1-Rgl	1.495 (±0.004)	0.405 (±0.003)	0.08 (±0.00)
	10	AdpGrd(30)	**1.488** (±0.007)	0.410 (±0.003)	8504.38 (±951.32)
		SmlOpt(30)	1.489 (±0.007)	0.410 (±0.003)	2189.76 (±478.19)
		FwdStep	1.509 (±0.007)	**0.416** (±0.003)	1.61 (±0.07)
		L1-Rgl	1.491 (±0.005)	0.415 (±0.002)	0.05 (±0.00)
Bike-D	5	AdpGrd(30)	0.996 (±0.011)	0.334 (±0.009)	1806.09 (±13.37)
		SmlOpt(30)	0.991 (±0.011)	**0.338** (±0.007)	146.13 (±6.46)
		FwdStep	**0.989** (±0.009)	0.335 (±0.008)	1.13 (±0.03)
		L1-Rgl	1.011 (±0.008)	0.319 (±0.008)	0.08 (±0.00)
	10	AdpGrd(30)	0.963 (±0.011)	**0.353** (±0.004)	6451.01 (±438.40)
		SmlOpt(30)	0.958 (±0.010)	**0.353** (±0.005)	1758.75 (±284.93)
		FwdStep	0.964 (±0.010)	0.349 (±0.006)	1.13 (±0.03)
		L1-Rgl	**0.956** (±0.011)	0.349 (±0.005)	0.05 (±0.00)

## Conclusion

This paper presented an MIO approach to sparse Poisson regression, which we formulated as an MIQO problem by applying piecewise-linear approximation to the nonlinear objective function. We also developed the adaptive greedy algorithm and the simultaneous optimization method to select a limited number of tangent lines that work well for piecewise-linear approximations.

We conducted computational experiments using synthetic and real-world datasets. Our methods for selecting tangent lines clearly outperformed conventional methods in terms of the quality of piecewise-linear approximations. For the synthetic datasets, our MIQO formulation delivered better prediction performance than did stepwise selection and *L*_1_-regularized estimation, especially in low-noise situations. Our MIQO formulation also compared favorably in terms of prediction performance with the other algorithms for real-world datasets.

Although our method can potentially find good-quality sparse regression models, applying it to large datasets is computationally expensive. It is more practical to choose between our method and heuristic algorithms according to the task at hand. We also expect our framework for piecewise-linear approximations to work well for various decision-making problems involving univariate nonlinear functions.

A future direction of study will be to develop an efficient algorithm specialized for solving our MIQO problem. We are now working on extending several MIO-based high-performance algorithms [24, 48, 49] to sparse Poisson regression. Another direction of future research is to improve the performance of our methods for selecting tangent lines. For example, although we selected tangent points of Eq (18) by evaluating each point *u*_*s*_ ∈ {−5.00, −4.99, −4.98, …, 4.99, 5.00} for *s* ∈ [*h*], tuning tangent points more finely will probably make marginal improvements in the prediction performance. In addition, to upgrade the prediction performance in high-noise situations, we should adopt the *L*_*p*_-regularization term *α*‖***w***‖_*p*_ with finely tuned parameters *α* and *p* in our MIQO formulation [50].
